# Detection of Failures in Metal Oxide Surge Arresters Using Frequency Response Analysis

**DOI:** 10.3390/s23125633

**Published:** 2023-06-16

**Authors:** Tiago Goncalves Zacarias, Rafael Martins, Carlos Eduardo Xavier, Julio Cezar Oliveira Castioni, Wilson Cesar Sant’Ana, Germano Lambert-Torres, Bruno Reno Gama, Isac Antonio dos Santos Areias, Erik Leandro Bonaldi, Frederico De Oliveira Assuncao

**Affiliations:** 1 R&D Department, Gnarus Institute, Itajuba 37500-052, MG, Brazil; tiagogz@live.com (T.G.Z.); germanoltorres@gmail.com (G.L.-T.); br.gama@unifei.edu.br (B.R.G.); isacareias@gmail.com (I.A.d.S.A.); erik@institutognarus.com.br (E.L.B.); fredeoa@gmail.com (F.D.O.A.); 2 Office of Research and Graduate Studies (PRPPG), Federal University of Itajuba, Itajuba 37500-903, MG, Brazil; 3 COPEL GeT, Curitiba 81200-240, PR, Brazil; rafael.martins@copel.com (R.M.); carlos.xavier@copel.com (C.E.X.); julio.castioni@copel.com (J.C.O.C.)

**Keywords:** condition monitoring, frequency response analysis, metal oxide surge arrester

## Abstract

This work presents an innovative application of Frequency Response Analysis (FRA) in order to detect early degradation of Metal Oxide Surge Arresters (MOSAs). This technique has been widely used in power transformers, but has never been applied to MOSAs. It consists in comparisons of spectra, measured at different instants of the lifetime of the arrester. Differences between these spectra are an indicator that some electrical properties of the arrester have changed. An incremental deterioration test has been performed on arrester samples (with controlled circulation of leakage current, which increases the energy dissipation over the device), and the FRA spectra correctly identified the progression of damage. Although preliminary, the FRA results seemed promising, and it is expected that this technology could be used as another diagnostic tool for arresters.

## 1. Introduction

A surge arrester is a widely used piece of equipment in an electrical power system, due to its capability of suppressing lightning or switching events [1,2,3]. Its correct operation guarantees the reliability of the system [4]. Until the end of the 1960s, the dominant technology in arresters was the use of a silicon carbide (SiC) varistor connected in series with a spark gap (whose function was to disconnect the varistor from the line after the surge). With the evolution of technology, the use of a zinc oxide (ZnO) varistor (with low impedance to the surge current) turned the use of the spark gap unnecessary. The ZnO varistor, actually, is the major component in the arrester, with other metal oxides used as minor additives in the intergranular spaces [5]—hence, the more general term Metal Oxide Surge Arrester (MOSA).

According to [6], moisture ingress was reported as the cause of 80% of outages in gapped arresters. In the case of sealed MOSAs, the humidity is usually low; although, after years of operation, the internal humidity can increase due to deterioration of the sealing. Hence, it is very important to monitor the condition of the arresters in service [7] before a catastrophic failure. The literature presents several propositions and improvements in monitoring methods that are briefly discussed below.

Monitoring methods for arresters can be divided between offline methods (where the device under test is removed from its energized operation) and online methods (where the device under test is monitored while still connected to the grid). The offline methods usually present more reliable diagnosis, although these methods may result in significant expenses of disassembly, transport and laboratory testing [8] (other than the cost of the interruption of service). According to [8], despite being less reliable, the online methods can provide a preliminary picture of the arrester condition. The online methods can highlight the monitoring of leakage current, the evaluation of temperature of the device and the monitoring of partial discharges.

The monitoring of leakage current is the most widely used method and it is the method with the largest number of variations in the literature. However, it relies on the measurement of the system voltage, as this is required in order to decompose the total leakage current into its capacitive and resistive components [9]. Reference [8] studied the influence of voltage fluctuations and their harmonics (3rd, 5th and 7th) on indicators, based on the harmonic content of the resistive leakage current. Reference [10] presents a methodology to extract the amplitude of the resistive leakage current without the use of the system voltage as a reference, based on statistics. Reference [11] evaluates the effects of ultraviolet aging and pollution on the harmonic content of the leakage current. Reference [9] presents the extraction of the harmonic contents of the total leakage current (without using the system voltage) based on a neural network.

Thermography is another popular method, due to its safe, easy and quick implementation. On the other hand, the elevated cost and the necessity for a specialist for the analysis are disadvantages [12]. Monitoring of arresters by thermography allows the identification of a rise in temperature, which is a result of modifications in certain characteristics of the arrester. Reference [13] presents a methodology of identification of the pollution degree on the arresters based on infrared thermography. The method of [13] uses artificial intelligence techniques in order to extract some features and perform a classification (comparing the performance of four typse of classifiers: K-nearest neighbor, Suport Vector Machine, Naïve Bayes and Random Forest). Other approaches based on artificial intelligence are presented in [14,15]. Reference [14] presents a system based on neuro-fuzzy networks (using input variables such as temperature, pollution index, constructive characteristics and some local measurements) in order to automatize the evaluation of thermographic pictures of arresters.

Some combinations of the previous methods have also been succesfully employed [15,16]. Reference [15] proposes thermal image temperature correlation with the 3rd harmonic’s resistive leakage current, based on an MLP neural network, in order to perform a classification of the condition of the arrester. Reference [16] also proposes a hybrid method, combining the leakage current with infrared imaging, based on a regression model.

As an alternative to the above online methods, one can highlight the methods which use the monitoring of partial discharges. According to [17], partial discharges can be measured based on electrical variables (such as current pulses measured with a high frequency current transformer [18]) or as non-electrical variables (such as sound [19], light [20] and electromagnetic waves [21]).

According to [8], offline methods (those which require interrupting operation) provide better insight into the arrester condition. The most-used offline methods are the reference voltage method, the power loss method and the insulation resistance method, which are briefly described bellow. These methods are commonly used in laboratories, due to limitations under operational conditions or other risks [17].

The reference voltage method is based on the application of a reference current (usually from 1 mA to 10 mA) and the measurement of the resulting voltage. Usually this test is performed before and after the application of a surge impulse (in the laboratory), as performed in [22]. If the reference voltage is decreasing, this is an indication of degradation (as less voltage would be required to generate the same reference current [23]).

The power loss method is based on the calculation of the dissipated power over the arrester [24]; hence, the synchronous measurement of the applied voltage and the leakage current is required (which is considered to be a disadvantage of the method [25]). The method, however, has been improved with the addition of an electrothermal model [26] and the operating history [27]. The use of the operating history allows the model to consider degradation caused by electrical and non-electrical factors observed in field operation.

The insulation resistance method is widely used in industry as a means to evaluate the condition of insulators. In normal operation, an arrester is an insulator; hence, the usual methods of insulation resistance can also be applied. These tests are performed with a megohmmeter, which applies a DC voltage (often elevated), and a small current is measured. In the case of arresters, the DC insulation test is considered to be a simple pass/fail test that produces results in only 60 s, though it is only suitable for low and medium voltage arresters [28].

This paper proposes a new methodology for monitoring the degradation of surge arresters, based on Frequency Response Analysis (FRA)—a technique that is widely used for diagnostic of power transformers and is based on varying some electrical parameters of the device being tested. According to [29], as a consequence of aging, there is a variation in several characteristic parameters of the ZnO varistor, including the dielectric parameters, which can, hence, be detected by FRA. Section 2 presents the aspects of theory required to understand the experiments of Section 3, including a brief description of the working principle of the zinc oxide surge arrester, its main parameters and the proposed method of diagnosis based on FRA. Section 3 presents some preliminary results on the offline application of FRA in order to detect degradation in arresters.

## 2. Materials and Methods

**Figure 1:**
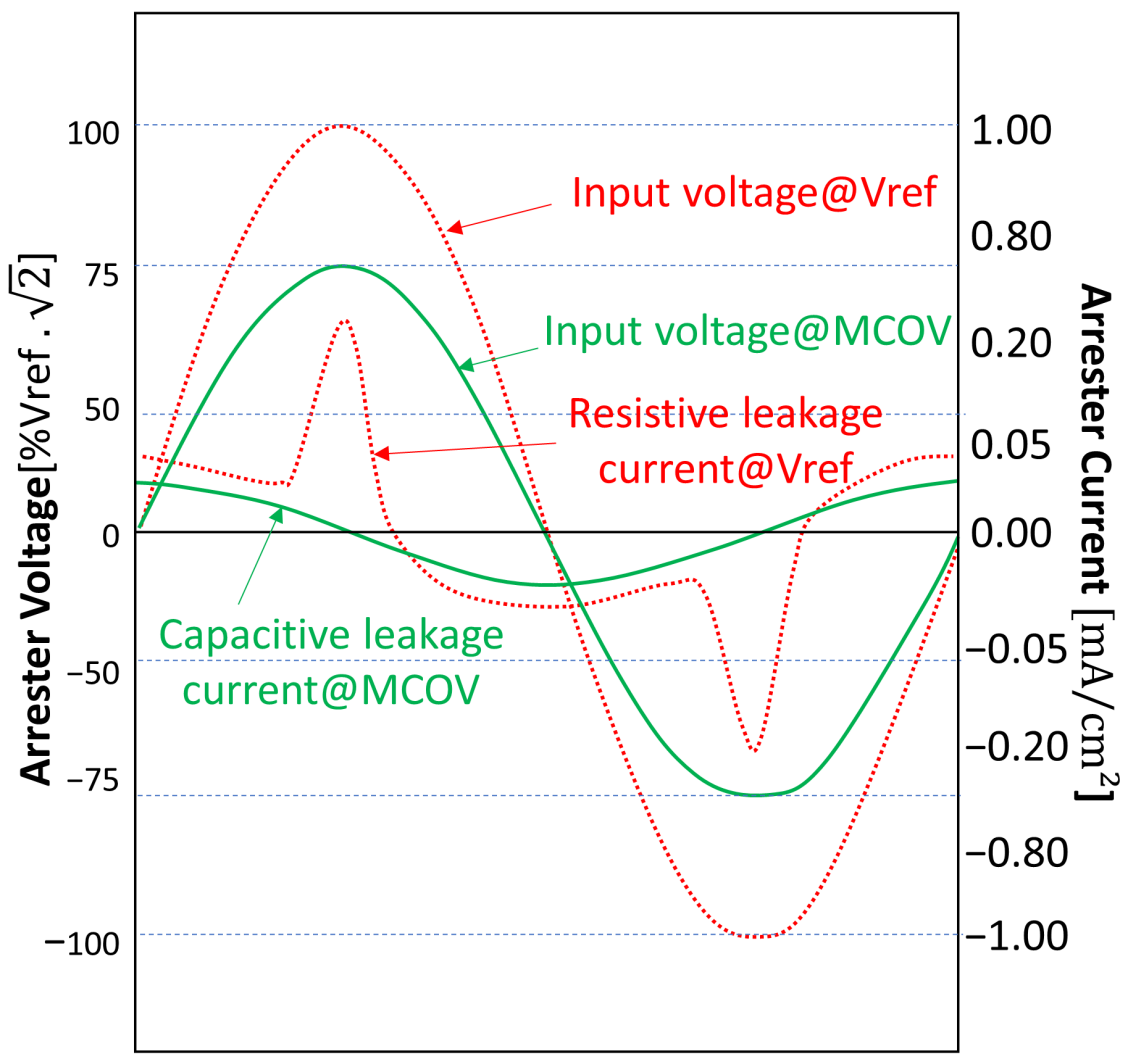
Parameters of voltage and current of ZnO arresters—based on [31].

The zinc oxide (ZnO) surge arrester has been introduced around 1976 and began to be widely used to protect electrical systems and their equipment against voltage surges. Due to its high non-linearity and higher energy absorption capacity, its discharge capability is more than twice the capability of the silicon carbide arrester with series gap (used prior to the ZnO arrester) [30]. Whenever unsolicited, the arrester must act as an insulator (with a small leakage current of a few miliamperes [30]). During a surge, it must carry an elevated current in order to reduce the voltage over the protected equipment. After the surge, it must return to the insulator condition. Figure 1 illustrates important parameters of the ZnO arresters, which are explained in the subsequent subsections.

### 2.1. Leakage Current

Even under normal operation, there is a small leakage current flowing through the arrester. This current is undesirable and, thus, a lower value is better. Under normal operation, the arrester acts as an insulator and this current has only a capacitive component (depicted at 90${}^{\circ }$, leading the applied voltage around peak MCOV in Figure 1).

Whenever the applied voltage is near the peak $V_{ref}$, a resistive component (depicted in phase with the applied voltage in Figure 1) is much greater than the capacitive component. Operation under this situation for more than 10 s results in an increase in energy dissipation, which starts to damage the arrester.

### 2.2. Maximum Continuous Operating Voltage (MCOV)

MCOV is defined by the IEEE Std C62.11 [32] as the maximum Root-Mean-Square (RMS) voltage that may be applied continuously between the terminals of the arrester. Under this voltage limit, the capacitive component of the leakage current is dominant.

### 2.3. Reference Voltage

Reference voltage is defined by the IEEE Std C62.11 [32] as the lowest peak value of voltage divided by square root of 2 (hence, the lowest RMS voltage) required to produce a resistive component in the leakage current high enough to dominate the capacitive current (in the range of 0.05 mA to 1 mA per square centimeter of disk area. In the case of the arrester used in the tests in Section 3 (with a disk area of 9.62 cm${}^{2}$), the reference current was considered to be in the range of 1 mA–10 mA.

### 2.4. Surge Arrester V×I Curve

**Figure 2:**
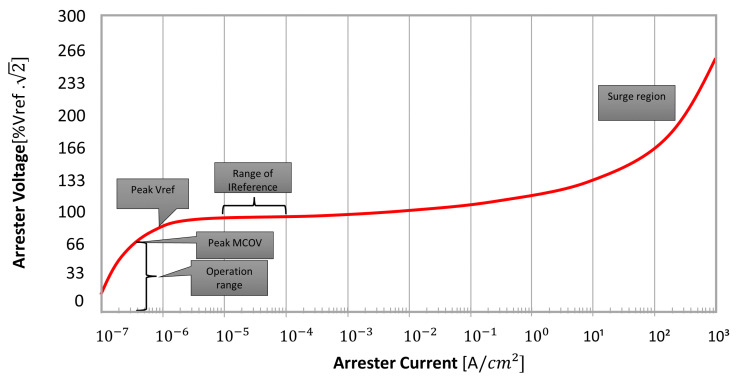
Typical *V×I* curve of ZnO arresters—based on [31].

Figure 2 presents a typical arrester voltage–current characteristic curve (*V×I* Curve), which relates the peak current that flows through the arrester for each respective applied voltage. This curve has three distinct regions:

1. Below the peak MCOV: region of normal operation, with only a small capacitive leakage current;
2. Just above the peak $V_{ref}$: region where the resistive leakage current dominates (with currents in the range of 0.05 mA–1 mA). The operation at this region must only be temporary, as the arrester is dissipating enough energy to trigger a thermal avalanche;
3. Surge: region where the arrester will start to conduct elevated currents caused by a surge of either switching or lightning.

### 2.5. Thermal Runaway

During prolonged exposure to a resistive leakage current, the arrester will start to dissipate energy, which raises the temperature of its internal disks. This, in turn, decreases its internal resistance and allows more current to flow, which raises the temperature even more—originating a thermal avalanche. This thermal avalanche is the end-of-life of the arrester.

### 2.6. Frequency Response Analysis

Frequency Response Analysis (FRA) has been widely used for offline diagnostics of transformers. For this purpose, it is a well-known technology and has several commercial equipment options available in the market [33]. Some other interesting applications of FRA have been proposed as well, such as condition monitoring of rotating machines [34] (although some issues with influence of rotor position have been reported in [35] and in [36]), detection of failures in tap changers [37] and detection of fluid degradation [38]. This current paper proposes an innovative use of FRA in order to detect degradation of ZnO surge arresters.

The FRA method can be summarized as the comparison of two spectra, measured at different instants of the lifetime of the device under test [39]. Differences between a present spectrum and its baseline spectrum (measured in the past, at a healthy condition) may indicate the onset of a failure.

**Figure 3:**
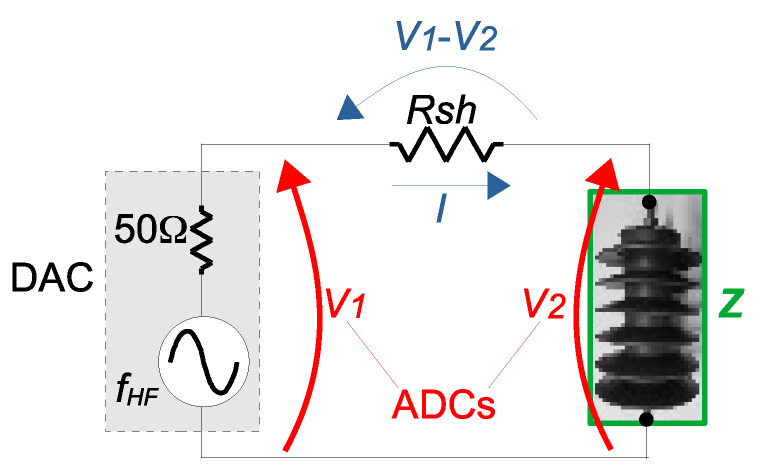
Measurement circuit for low-cost FRA setup, using general purpose DAC and ADCs and a shunt resistor.

Reference [40] presents a step-by-step guide on the implementation of a low-cost system to obtain FRA spectra, using a Zynq ARM–FPGA board. This same system has been employed in some of the experimental tests in Section 3, although any other low-cost FRA or commercial system for FRA of transformers could be used. In the case of the low-cost system of [40], one programmable Digital-to-Analog Converter (DAC) of the ARM–FPGA board is used to generate the frequency sweep to the measuring circuit, as presented in Figure 3. Two general purpose Analog-to-Digital Converters (ADC) of the board are used to measure the DAC voltage ($V_{1}$) and the voltage across the arrester under test ($V_{2}$). The current flowing through the arrester under test is measured indirectly using a shunt resistor ($R_{sh}$). Based on the amplitude of the signal $V_{2}$ ($\left|{\vec{V}}_{2}\right|$) and on the amplitude of the signal resulting from the subtraction of $V_{2}$ from $V_{1}$ ($\left|{\vec{V}}_{1}-{\vec{V}}_{2}\right|$), the magnitude of the impedance *Z* of the arrester can be determined as Equation (1) [33]. Also, based on the phase of the signal $V_{2}$ ($∠{\vec{V}}_{2}$) and on the phase of the signal resulting from the subtraction of $V_{2}$ from $V_{1}$ ($∠\left({\vec{V}}_{1}-{\vec{V}}_{2}\right)$), the phase angle of the impedance *Z* ($∠\vec{Z}$) of the arrester can be determined as Equation (2). The amplitude and phase of the signals ${\vec{V}}_{2}$ and $\left({\vec{V}}_{1}-{\vec{V}}_{2}\right)$ can be extracted using a DFT [41].

(1) $$\left|\vec{Z}\right|=\frac{\left|{\vec{V}}_{2}\right|}{\left|\vec{I}\right|}=\frac{\left|{\vec{V}}_{2}\right|}{\left|\frac{{\vec{V}}_{1}-{\vec{V}}_{2}}{R_{sh}}\right|}=R_{sh}\cdot \frac{\left|{\vec{V}}_{2}\right|}{\left|{\vec{V}}_{1}-{\vec{V}}_{2}\right|}\;.$$

(2) $$∠\vec{Z}=∠{\vec{V}}_{2}-∠\vec{I}=∠{\vec{V}}_{2}-∠\left({\vec{V}}_{1}-{\vec{V}}_{2}\right)\;.$$

Equations (1) and (2) perform a calculation of one point at the magnitude spectrum and one point at the phase spectrum (respectively) for the specific frequency $f_{HF}$ being injected by the DAC. Then, the frequency is incremented and the spectra are obtained. Each spectrum is compared against its baseline, in order to detect a discrepancy (which might imply in change in some of the properties of the arrester under test).

It has to be noted that the value of the shunt resistor has an effect on the measurements. Its ideal value should be of the same order as the impedance being measured. Considering the output of the DAC as 0.7 $V_{peak-to-peak}$ and an $R_{sh}$ = 1 kΩ (such as the one used in Section 3.1), the measurement range is from 10 Ω to less than 1 MΩ. The upper limit can be further extended to a few MΩ with the use of an amplifier (in the case of Section 3.1, a gain of 10 was used, resulting in a voltage of 7 $V_{peak-to-peak}$). According to [42], in order to maintain repeatability in the results, the use of the same setup is recommended in in all measurements (which includes the value of the shunt resistor and the use of the amplifier).

In the case of commercial FRA equipment, usually, the input impedance of the ADCs is 50 Ω. Additionally, usually, the spectra obtained with commercial FRA equipment are in the form of a gain (in dB) over frequency. In the case of FRA in power transformers, this gain is a ratio between the voltages measured at two different terminals (either of the same winding or with respect to another winding). This type of configuration has the advantage of being independent of the shunt resistor. However, the resulting spectra only represent a gain between two voltages and not an impedance. In the case of FRA of transformers, the main problems detected are related with deformations and displacements in the windings (although some electrical insulation problems, such as shorted turns and core grounding issues, are also detected). However, in the case of arresters, it makes more sense to analyze the variation in impedance (at least, in these preliminary tests), as the goal is to observe the degradation of the semiconductor insulation. Depending on the manufacturer of the commercial FRA equipment, some different configurations can be used. Specifically, for the *Bode-100* (of manufacturer OMICRON Lab, Klaus, Austria) (used in the tests in Section 3.2), an interesting configuration for arresters is the so-called “Series-Thru” (with voltage output of 0 dBm = 0.63 $V_{peak-to-peak@50\Omega }$), allowing impedance measurements in the range of 1 kΩ to a few MΩ [43]. With the addition of a 12 dB amplifier accessory (model *B-AMP 12*, resulting in a voltage output of 12 dBm = 2.52 $V_{peak-to-peak@50\Omega }$), the upper limit can be further extended to tens of MΩ.

## 3. Results and Discussion

**Figure 4:**
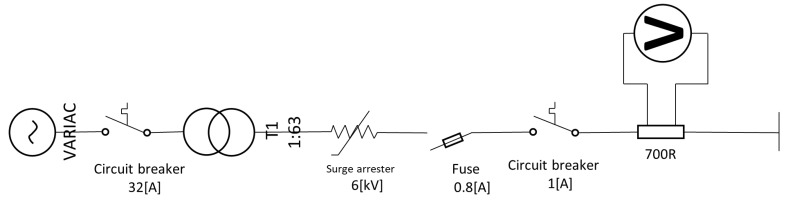
Schematic circuit of the accelerated aging test setup.

**Figure 5:**
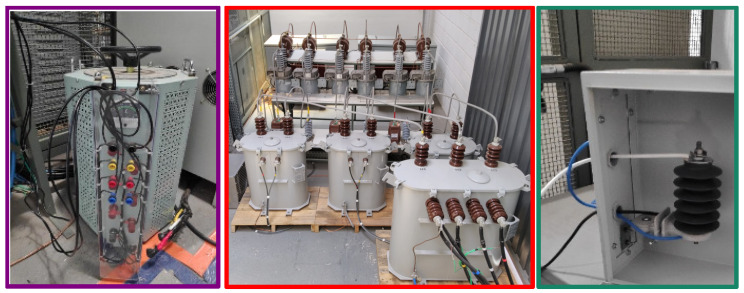
Photograph of the accelerated aging test setup (low voltage VARIAC—medium voltage test substation—arrester that was aged).

Figure 4 presents the schematic circuit used to perform the accelerated aging tests on the arrester samples. In order to increase the voltage above the MCOV of the arrester, a 220V VARIAC (of manufacturer JNG, Wenzhou, China) was connected at the low voltage windings of a 220 V:13.8 kV (1:63) transformer. The arrester was connected to the medium voltage windings, with a shunt resistor of 700 Ω (in order to measure the leakage current with a voltmeter). Circuit breakers and a fuse were also included for protection, in case of accidental conduction of the arrester. Figure 5 presents a photograph of the medium voltage test substation (previously presented in [44]), illustrating where the 1:63 transformer was located and where the test could be conducted safely. The figure also presents the low voltage VARIAC (located outside the protective fence) and the arrester that was aged (located inside the fence).

Two groups of tests were performed. In the first group of tests (presented in Section 3.1), the samples were brought to failure condition and some parameters were measured. In the second group of tests (presented in Section 3.2), the degradation of the samples was slowly increased, in order to see the evolution of the measured parameters according to the degradation level. In both cases, through the VARIAC in Figure 5, a voltage higher than the MCOV was applied (in order to force a leakage current). The difference was the duration of exposure to the leakage currents. After each exposure to leakage current, the arrester was tested using a megohmmeter (which is one of the offline tests described in the literature, and the easiest to be performed without complex laboratory equipment) and some frequency response methods (using an LCR meter and the FRA). In all tests, the results showed agreement with the damage level (presenting, simultaneously, a decrease in the insulation resistance and an increase in resistive losses as the impedance angle shifted from capacitive ($-90^{\circ }$) towards resistive ($0^{\circ }$) behavior).

### 3.1. Analysis of Failure

The failure analysis consisted of evaluating changes in the physical and electrical characteristics of the internal disks of test samples of surge arresters. The test consisted of forcing a high leakage current through the samples for two cycles of 8 h (each cycle).

Two samples (identified as *PR-4* and *PR-5*) were exposed to a voltage (7.4 kV${}_{RMS}$) higher than the their MCOV (5.1 kV${}_{RMS}$, in order to force a leakage current of around 10 to 15 mA. The prolonged permanence of the samples under this condition triggers a thermal avalanche, which culminates in the failure of the samples.

**Figure 6:**
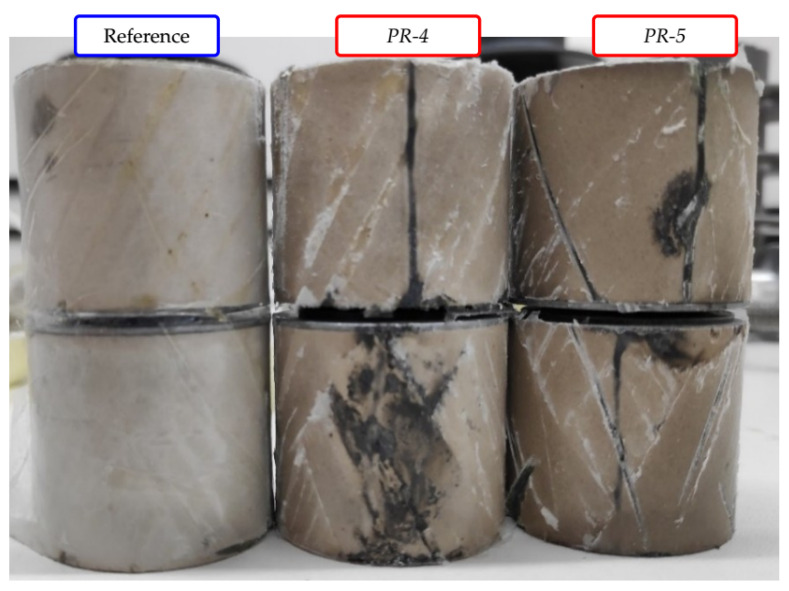
Disassembled samples after prolonged exposure to leakage current.

**Figure 7:**
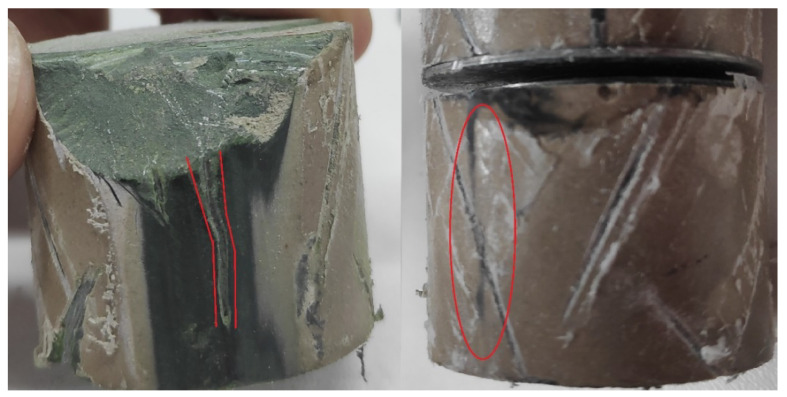
Detailed surface tracking on sample *PR-5*.

After the test, both samples were disassembled. Additionally, a brand new sample (which did not suffer any damage) was also disassembled, to serve as a visual reference. Figure 6 presents the three disassembled samples. It can be noted that the thermal avalanche (combined with the high voltage in the test) caused some surface tracking and puncture at both *PR-4* and *PR-5*. Sample *PR-5* was further disassembled, in order to better observe the surface tracking, as presented in Figure 7.

The insulation resistances of the samples (before and after the failure) were measured (with a megohmmeter, under 2.5 kV and 5 kV), as presented in Table 1. The low insulation resistance measured after the prolonged exposure to leakage current is an indication of failure of both samples.

An LCR meter was also used (before and after the test), in order to observe changes in some electrical parameters of the samples, as presented in Table 2. It can be noted that the samples changed from a capacitive impedance ($\theta \approx -90^{\circ }$, characteristic of an insulator material) before the failure towards a resistive impedance ($\theta \approx 0^{\circ }$) after the failure.

**Figure 8:**
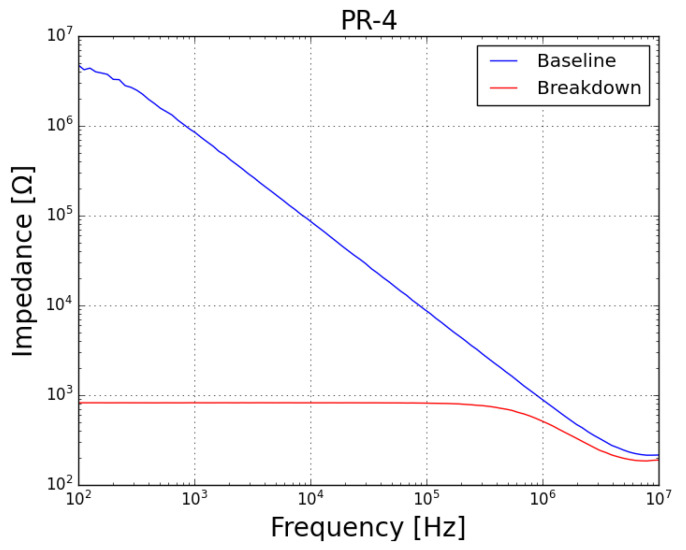
Magnitude plot of FRA sweep on sample *PR-4* at baseline condition (blue plot) and after failure by prolonged exposure to leakage current (red plot).

**Figure 9:**
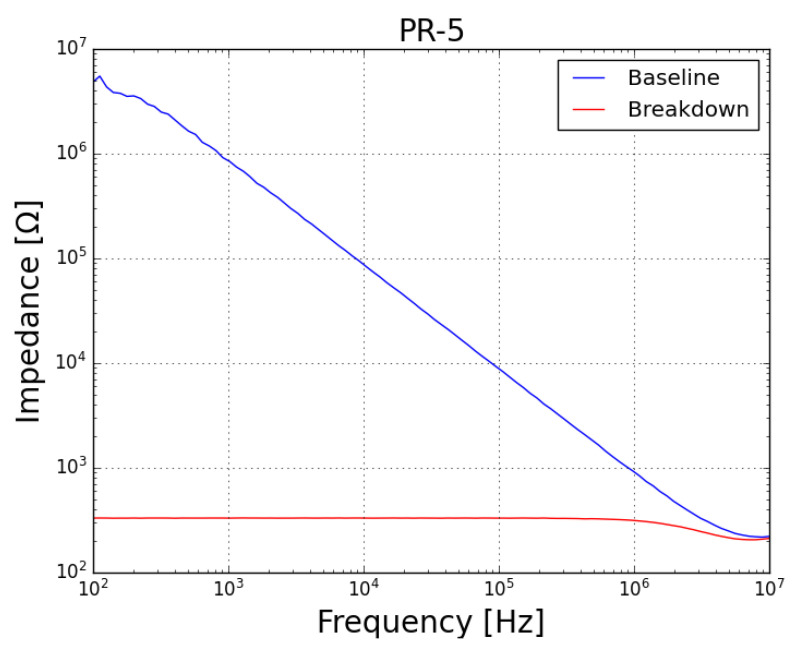
Magnitude plot of FRA sweep on sample *PR-5* at baseline condition (blue plot) and after failure by prolonged exposure to leakage current (red plot).

As well, an FRA sweep (obtained with the prototype described in [40], with a shunt resistor $R_{sh}$ = 1 kΩ, amplification by a factor of 10 at the DAC and attenuation of 0.1 at the ADCs) was performed on the samples, both before (blue plots) and after (red plots) the test. Concerning the impedance (magnitude) spectra of both samples (*PR-4* presented in Figure 8 and *PR-5* presented in Figure 9), it can be noted that, before the test, the samples behaved as a capacitor (with reactance dropping with the increase in frequency, which is characteristic of a good insulator) and, after the failure, both samples behaved as a resisistor (constant reactance, independent of frequency).

**Figure 10:**
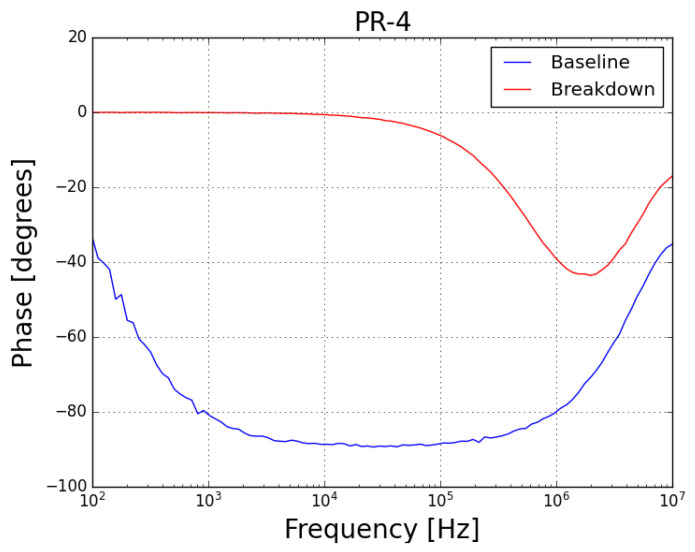
Phase plot of FRA sweep on sample *PR-4* at baseline condition (blue plot) and after failure by prolonged exposure to leakage current (red plot).

**Figure 11:**
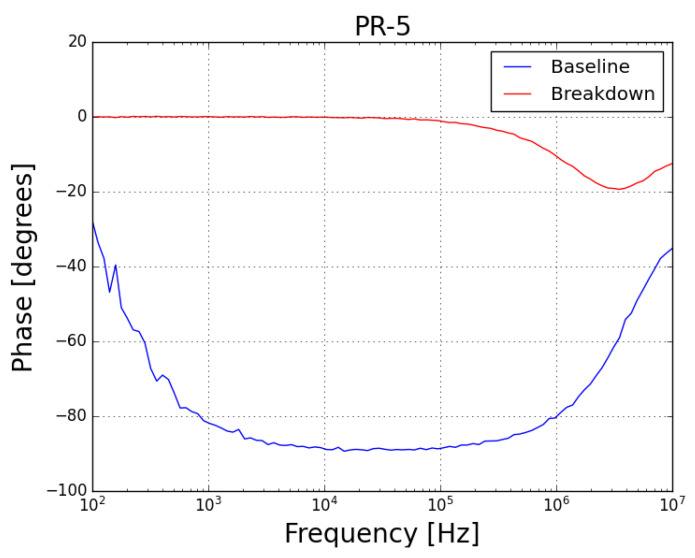
Phase plot of FRA sweep on sample *PR-5* at baseline condition (blue plot) and after failure by prolonged exposure to leakage current (red plot).

Concerning the phase spectra of both samples (*PR-4* presented in Figure 10 and *PR-5* presented in Figure 11), it can be noted that, before the test, the samples behaved as a capacitor (with phase angle near 90${}^{\circ }$, which is characteristic of a good insulator) and, after the failure, both samples behave as a resistor (with phase angle near 0${}^{\circ }$).

### 3.2. Analysis of Slow Degradation

**Figure 12:**
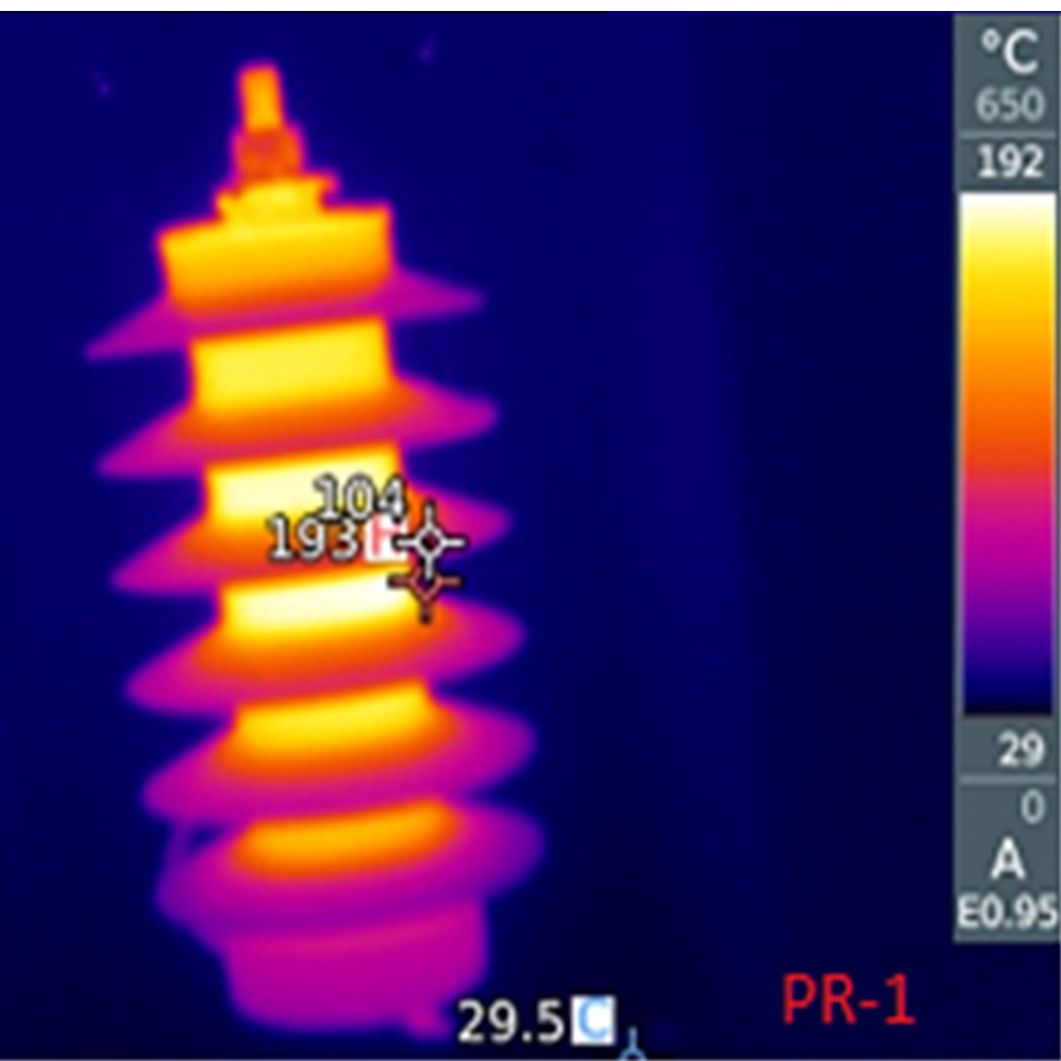
Monitoring of temperature during each cycle.

Three samples (identified as *PR-1*, *PR-2* and *PR-6*) were exposed to a voltage (7.4 $kV_{RMS}$) higher than the their MCOV (5.1 $kV_{RMS}$), in order to force a leakage current of around 10 to 15 mA (justas in Section 3.1). The permanence (for cycles with duration of around 60 min) of the samples under this condition triggers a thermal avalanche, which gradually produces a degradation of the samples. Figure 12 presents an infrared photograph of one of the samples during a cycle. During the cycles, the temperatures reached values superior to 170 ${}^{\circ }$C.

After the samples cooled, a series of electrical measurements were performed (for each cycle). Table 3, Table 4 and Table 5 present the values of the measurements for each of the samples after three cycles of thermal degradation. The first line of each table presents the measurements for the unstressed samples, in order to serve as a baseline. In all cases, there were measurements of the insulation resistance (performed with a megohmmeter) and measurements of impedance (magnitude and phase at 100 Hz) performed with an LCR meter.

For the three samples, it can be noted that the impedances gradually changed from capacitive ($\theta \approx -90^{\circ }$, characteristic of an insulator material) towards a more resistive behavior ($\theta \to 0^{\circ }$). Also, after each cycle, the insulation resistance decreased in value.

**Figure 13:**
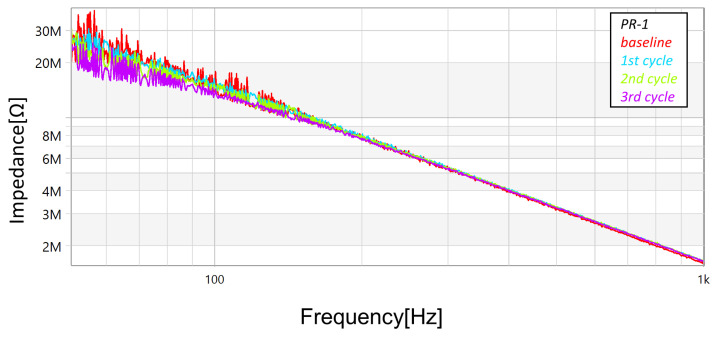
Progression of damage on sample *PR-1*, detected using FRA (magnitude spectra).

**Figure 14:**
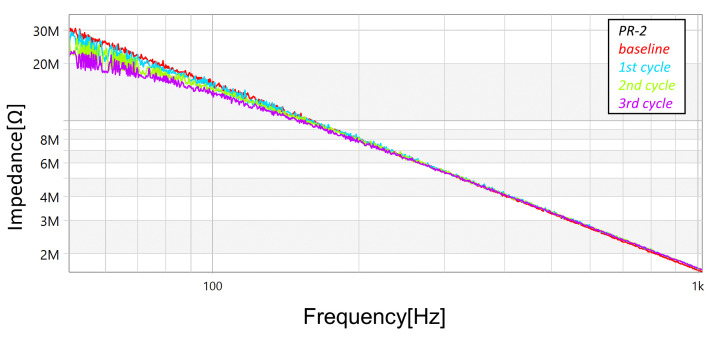
Progression of damage on sample *PR-2*, detected using FRA (magnitude spectra).

**Figure 15:**
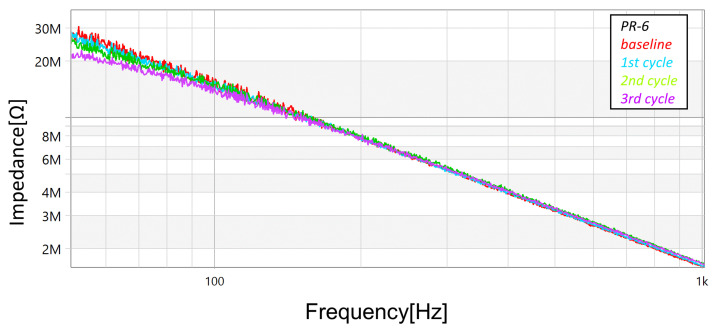
Progression of damage on sample *PR-6*, detected using FRA (magnitude spectra).

In addition to the measurements with the megohmmeter and the LCR meter, FRA sweeps (obtained using commercial equipment—OMICRON’s BODE100, in the “Series-Thru” configuration and with the 12 dB amplifier accessory) were performed after each cycle (and also at the unstressed baseline condition). Figure 13, Figure 14 and Figure 15 present the magnitude responses of the FRA sweeps for the three samples. Over the whole range of frequencies, there is a visible a trend in the measurements—the gradual reduction in magnitude as the stresses progress—although, the trend is more evident in the frequency range below 100 Hz.

**Figure 16:**
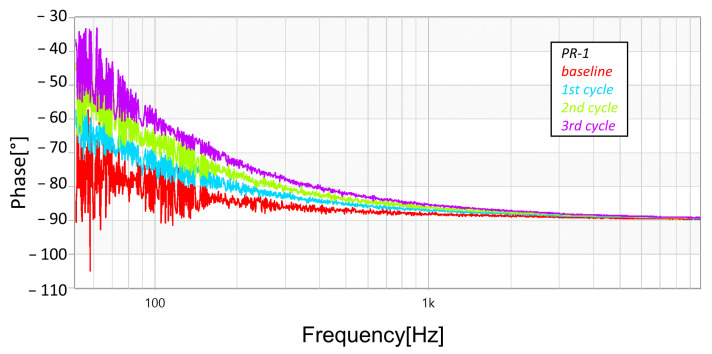
Progression of damage on sample *PR-1*, detected using FRA (phase spectra).

**Figure 17:**
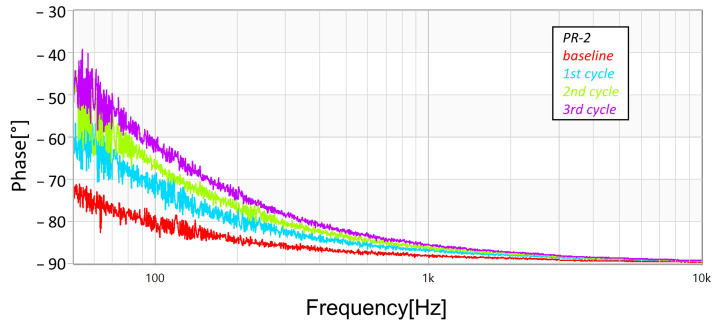
Progression of damage on sample *PR-2*, detected using FRA (phase spectra).

**Figure 18:**
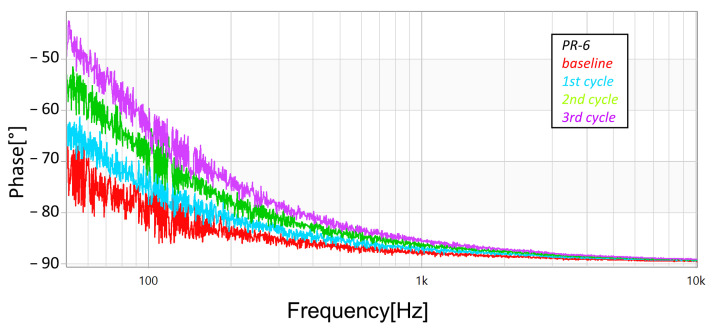
Progression of damage on sample *PR-6*, detected using FRA (phase spectra).

As well, Figure 16, Figure 17 and Figure 18 present the phase responses of the same FRA sweeps of Figure 13, Figure 14 and Figure 15. It is very interesting to note that the phase spectra are much more responsive than the magnitude spectra. This represents a high potential to detect more subtle damage on the arresters.

## 4. Conclusions

This paper presented the application of Frequency Response Analysis (FRA) in order to detect degradation in metal oxide surge arresters. The FRA technique is a widely used method for detection of damage in power transformers (with some other applications as well). This paper investigated its use in surge arresters—which constitutes an innovative use. The results presented in this paper (still preliminary) have shown that FRA is capable of detecting early damage on the arresters (caused by a controlled circulation of leakage current and the consequent power dissipation over them). Both magnitude and phase spectra were able to distinguish between the levels of degradation, although it was very interesting to notice that the phase spectra were more responsive to the levels of degradation than the magnitude spectra. This finding presents a high potential to detect more subtle degradation on the arresters, although more investigation needs to be performed in other cases.

It is important to note that the degradation tests performed were accelerated tests, where the arrester was stressed beyond some threshold in order to produce a degradation. It is reasonable to question if, under normal operation, a very incipient degradation could be detected. The sensitivity of the FRA to these incipient damages (as well as modeling a relationship between a certain type of failure with a deviation pattern in the FRA spectra) is a matter to be studied in future work. However, as the aging of the arrester results in variations in its electrical parameters [29] and the FRA method is based on the detection of variations in electrical parameters, one can conclude that FRA is suitable for detection of aging in arresters. Along with the deterioration by leakage current, other types of deterioration tests, such as moisture ingress, contaminants, pollution, UV radiation, etc., have to be performed and tested with the FRA method.

Also, as future work, it is envisaged to adapt the FRA (which is essentially an offline method) in order to detect early damage online (without the necessity to remove the arrester from service). The main issue concerning online operation is the supply voltage, which may damage the electronics of the FRA equipment. The literature on the use of FRA on electric machinery (transformers, motors and generators) has presented some efforts related to this adaptation (using a coupling system, either passive [45] or active [33]). In both cases, the coupling system acts as a high pass filter, allowing the high frequency of the FRA to be injected into the device under test and, at the same time, blocking the supply voltage to avoid damage to FRA electronics. While these alternatives may be considered for medium voltage distribution systems, the application in high voltage transmission systems might require other solutions. Additionally, other limitations for online operation still have to be considered, such as the potential disconnection of the FRA equipment during a lightning or switching surge.

## Figures and Tables

**Table 1:** Insulation resistance before and after failure by prolonged exposure to leakage current.

Sample	Megohmmeter Voltage	Insulation Resistance	
		Before Failure	After Failure
*PR-4*	2.5 kV	51.0 GΩ	<1 kΩ
	5 kV	9.3 GΩ	<1 kΩ
*PR-5*	2.5 kV	52.1 GΩ	<1 kΩ
	5 kV	10.2 GΩ	<1 kΩ

**Table 2:** Impedance (before and after failure by prolonged exposure to leakage current) measured with an LCR meter at 100 Hz.

Sample	LCR m @ 100 Hz	
	Before Failure	After Failure
*PR-4*	15.98 MΩ $∠-87.614^{\circ }$	817.8 Ω $∠-0.0047^{\circ }$
*PR-5*	16.92 MΩ $∠-87.33^{\circ }$	248.6 Ω $∠-0.0126^{\circ }$

**Table 3:** Measurements performed for sample *PR-1*.

Cycles	Megohmeter (1 kV)	LCR @ 100 Hz
unstressed (baseline)	19.2 GΩ	15.6 MΩ $∠-88.08^{\circ }$
1st cycle	87.1 MΩ	15.72 MΩ $∠-79.3^{\circ }$
2nd cycle	42.5 MΩ	14.96 MΩ $∠-70.6^{\circ }$
3rd cycle	27.7 MΩ	14.56 MΩ $∠-66.78^{\circ }$

**Table 4:** Measurements performed for sample *PR-2*.

Cycles	Megohmeter (1 kV)	LCR @ 100 Hz
unstressed (baseline)	135 GΩ	15.93 MΩ $∠-88.08^{\circ }$
1st cycle	91.1 MΩ	15.8 MΩ $∠-79.4^{\circ }$
2nd cycle	53.5 MΩ	15.4 MΩ $∠-73.2^{\circ }$
3rd cycle	33.1 MΩ	14.67 MΩ $∠-66.7^{\circ }$

**Table 5:** Measurements performed for sample *PR-6*.

Cycles	Megohmeter (1 kV)	LCR @ 100 Hz
unstressed (baseline)	7.9 GΩ	15.93 MΩ $∠-88.08^{\circ }$
1st cycle	124 MΩ	15.8 MΩ $∠-81.42^{\circ }$
2nd cycle	64.1 MΩ	15.4 MΩ $∠-77.7^{\circ }$
3rd cycle	32.8 MΩ	14.79 MΩ $∠-70^{\circ }$

## Data Availability

Data sharing is not applicable to this article.

## Abbreviations

The following abbreviations are used in this manuscript:

ADC	Analog-to-Digital Converter
DAC	Digital-to-Analog Converter
DFT	Discrete Fourier Transform
FRA	Frequency Response Analysis
FPGA	Field Programmable Gate Array
MCOV	Maximum Continuous Operating Voltage
MLP	MultiLayer Perceptron
MOSA	Metal Oxide Surge Arrester
ZnO	Zinc Oxide
RMS	Root Mean Square
SiC	Silicon Carbide
